# The Role of Immunotherapy in the First-Line Treatment of Elderly Advanced Non-Small Cell Lung Cancer

**DOI:** 10.3390/cancers15082319

**Published:** 2023-04-15

**Authors:** Alessia Spagnuolo, Cesare Gridelli

**Affiliations:** Division of Medical Oncology, ‘S. G. Moscati’ Hospital, 83100 Avellino, Italy

**Keywords:** elderly patients, geriatric oncology, immune checkpoint inhibitors, immunotherapy, NSCLC, older patients, toxicity

## Abstract

**Simple Summary:**

The treatment of advanced non-small cell lung cancer (NSCLC) after the age of 65 raises age-related problems as the elderly are often affected by other diseases, not infrequently chronic, take drugs that may interfere with anti-cancer treatment and are sometimes unable to fully understand relevant information. Moreover, they do not represent the ideal patient for enrolment in clinical trials, even with immunotherapy, which is now the choice therapy for the first-line treatment of NSCLC. With a view to offering more time to patients with metastatic NSCLC, the issues of quality of life and appropriateness of oncological care in the elderly are of primary importance. This review stresses the need to find a common approach to lung cancer management in a steadily aging society and describes the main currently available data on the use of immune checkpoint inhibitors in older patients with advanced NSCLC, confirming the necessity for reliable biomarkers that predict immune response to assess which patients benefit from which type of immunotherapy.

**Abstract:**

Immune checkpoint inhibitors have changed the history of NSCLC treatment by becoming, alone or in combination with platinum-based chemotherapy, a mainstay of first-line therapy for advanced NSCLC. This increasingly dictates the identification of predictive biomarkers of response that can guide patient selection, in order to rationalize and personalize therapies, particularly in elderly patients. Immunotherapy in these patients raises questions of efficacy and tolerability related to aging, which is accompanied by a progressive decline in various body functions. Physical, biological and psychological changes contribute to individual validity status and, preferably, ‘fit’ patients are generally enrolled in clinical trials. In elderly patients, especially frail and complex patients with more than one chronic disease, data are poor and specific prospective studies are needed. This review reports the main available results on the use of immune checkpoint inhibitors in older patients with advanced NSCLC, in terms of efficacy and toxicity, and aims to highlight the need to better predict which patients might benefit from immunotherapy agents by probing knowledge and integrating information on immune system changes and age-related physiopathological modifications.

## 1. Introduction

Lung cancer is one of the most common oncological diseases. For a long time, in fact, the diagnoses of lung cancer have exceeded those of other neoplasias at the global level. Nowadays, over 2.2 million cases are diagnosed each year worldwide. Unfortunately, its mortality remains the highest among all the cancers: every year there are almost 1.8 million deaths globally due to lung cancer [1]. Thanks to research, however, today it is possible to achieve an accurate and personalized diagnosis and many available therapeutic innovations, such as immunotherapy, are able to increase both survival and the quality of patients’ lives. The proportion of elderly patients in the global lung cancer population is steadily increasing and the age range in which this cancer is most frequently diagnosed is 65–74 years [2]. In parallel, this is also the age group with the highest percentages of deaths due to lung cancer [2]. However, there is less scientific evidence concerning the treatment for lung cancer in elderly patients, both from a quantitative and qualitative point of view, than in younger patients, even if in recent years a greater awareness of the problem has led to more clinical research in this field. Non-small cell lung cancer (NSCLC) represents approximately 85% of lung cancers and the majority are diagnosed at advanced stages [2,3]. This review aims to describe and analyze in depth the current role of frontline immunotherapy, alone or in combination with chemotherapy, in the fight against advanced NSCLC in the elderly patient.

## 2. Who Are the Elderly Patients?

Elderly patients with cancer, including NSCLC, represent a distinct population in which the treatment and prevention of side effects caused by anti-cancer therapy assume a particular relevance for two reasons. First, the lower tolerability of side effects results in a need for adequate support and, secondly, there is a greater risk of pharmacological interference due to the simultaneous intake of multiple drugs deriving from the high prevalence of comorbidities at an older age. For the definition of “elderly”, considering that over half of cancer patients are 65 or older, the most frequently used cutoff in clinical studies is the age of 65 years [4,5], although in some trials the age of 70 is the upper limit [6,7]. It is a common opinion that a chronological cutoff to define the elderly patient is not the most appropriate and that the identification of the individual’s biological rather than chronological age would be preferable. This would allow better planning of adequate medical treatment in the elderly [8], and more specifically in patients affected by NSCLC, where immunotherapy has marked a turning point. An important aspect related to age and the use of immunotherapy drugs is the phenomenon of immunosenescence, i.e. the decline of the individual’s immune capacity [9]. This could evidently limit the efficacy of immune checkpoint inhibitors (ICIs) and influence the tolerability of the treatment [10]. More specifically, alterations of physiology, polytherapy, loss of functional integrity, reduction of social support and limited economic resources contribute to the patient’s performance status (PS), which certainly represents an essential prognostic factor for lung cancer, although it is not able to accurately predict outcome in elderly patients. A multidimensional geriatric assessment that takes into account not only comorbidities but also functional, mental and nutritional status has been adopted in order to evaluate elderly cancer patients. This can be useful for selecting individuals who are adequate and more likely to benefit from standard treatment, compared to those who are vulnerable and need individualized care, or are frail and are candidates for supportive care only [11]. Evidence from the scientific literature regarding the efficacy of immunotherapy as first-line treatment in elderly patients with advanced NSCLC is shown below.

## 3. Immunotherapy in Elderly Patients

First-line immunotherapy treatment options for advanced NSCLC include: single-agent ICI (pembrolizumab, atezolizumab, cemiplimab), ICI (pembrolizumab, atezolizumab, cemiplimab)—chemotherapy combinations, and ICI-ICI (nivolumab plus ipilimumab, durvalumab plus tremelimumab)—chemotherapy combinations.

### 3.1. Mono-Immunotherapy

KEYNOTE-024 is a phase III controlled study for the treatment of previously untreated metastatic NSCLC. Patients with tumor proportion score (TPS) ≥ 50% and an Eastern Cooperative Oncology Group (ECOG) PS 0–1 were included. They were randomized to receive pembrolizumab (anti-programmed death-1 (PD-1) monoclonal antibody) or platinum-containing chemotherapy according to tumor histotype (squamous or non-squamous). In the updated analysis, a hazard ratio (HR) (95% confidence interval (CI)) of 0.60 (0.38–0.96) in overall survival (OS) in the subgroup of patients aged <65 years, and a HR of 0.64 (0.42–0.98) in the subgroup of patients aged ≥65 years were reported with immunotherapy treatment [12]. Similarly, treatment with pembrolizumab resulted in comparable OS outcomes between patients older and younger than 65 with PD-L1-expressing, advanced and untreated NSCLC in the randomized phase III study KEYNOTE-042 [13]. The pooled analysis of studies KEYNOTE-010, -024, and -042 was conducted to evaluate the efficacy and safety of pembrolizumab monotherapy versus chemotherapy in elderly patients (≥75 years) with advanced, PD-L1 positive NSCLC [14]. The study included 264 elderly patients from KEYNOTE-010 (*n* = 90), -024 (*n* = 45) and -042 (*n* = 129) intent-to-treat populations and 2348 patients younger than 75 years of age. Half of the patients in the ≥75 years age subgroup had a PD-L1 ≥ 50% (132/264). Pembrolizumab monotherapy reduced the risk of death by 60% in patients ≥ 75 years of age and PD-L1 ≥ 50% (HR 0.40, 95% CI 0.25–0.64) and resulted in a longer median OS of 23.1 months (11.9—not reached), versus 8.3 months (7.0–11.1) with chemotherapy. Pembrolizumab monotherapy reduced the risk of death by 33% in patients < 75 years of age and PD-L1 ≥ 50% (HR 0.67, 95% CI 0.57–0.78) and resulted in a longer median OS of 19.2 months (16.4–22.4) compared to 11.9 months (10.1–13.1) with chemotherapy (Table S1A) [14]. Another pooled analysis of eight randomized trials of first-line immunotherapy combined with chemotherapy or alone showed similar results among available treatment options in patients 75 years of age or older (HR 0.95, 95% CI 0.42–2.14) (Table S1B) [15].

IMpower 110 is a phase III randomized study comparing atezolizumab (anti-PD-L1) monotherapy versus chemotherapy in chemotherapy-naïve patients with non-squamous or squamous stage IV NSCLC selected on the basis of PD-L1 expression. Atezolizumab increased OS by 7.1 months compared with chemotherapy (median OS 20.2 versus 13.1 months; HR 0.59, 95% CI 0.40–0.89; *p* = 0.0106) in patients with elevated PD-L1 expression (tumor cell (TC)3 o tumor-infiltrating immune cell (IC)3-wild-type). In the subgroup of patients 65–74 years of age, HR was 0.63 (95% CI 0.34–1.19) while among the 23 patients (11.2%) over 74 years of age it was 0.79 (95% CI 0.18–3.56) [16].

IPSOS is a phase III randomized study involving untreated patients with locally advanced/metastatic squamous or non-squamous NSCLC. It investigated the effects of atezolizumab (2:1 allocation) compared with that of mono-chemotherapy in more than 450 patients who were not candidates for platinum-based chemotherapy. Reasons for non-eligibility for platinum included an ECOG PS ≥ 2 or an age of 70 years and above and relevant comorbidities. The mean age of the participants was 75 years and nearly a third were 80 or older, while 83% had an ECOG PS of 2 or higher [17]. With a median follow-up of 41.0 months, atezolizumab led to a median OS of 10.3 months (95% CI 9.4–11.9) versus 9.2 months (95% CI 5.9–11.2) achieved with chemotherapy (stratified HR 0.78, 95% CI 0.63–0.97; *p* = 0.028). The 24-month OS rate with atezolizumab was approximately double that obtained with chemotherapy (24.3% and 12.4% respectively). In addition to achieving the primary endpoint, the results also showed a double objective response rate (ORR) in the immunotherapy arm compared to the comparator arm: 16.9% (95% CI 12.8–21.6%) versus 7.9% (95% CI 4.2–13.5%); median duration of response (DOR) was also almost doubled with atezolizumab: 14.0 months (95% CI 8.1–20.3) against 7.8 months (95% CI 4.8–9.7) [17]. From a quality life point of view (QoL), valuated by means of the European Organisation for Research and Treatment of Cancer (EORTC) QLQ-C30 and QLQ-LC13 functional scales and symptom questionnaires, it was observed that cognitive, social and role functions remained stable or improved during week 48 of atezolizumab treatment compared to baseline. Though not estimable in either arm, the median time to worsening of chest pain indicated a more favorable HR for the arm treated with the anti-PD-L1 (HR 0.51, 95% CI 0.27–0.97). 57% of patients in the atezolizumab arm and 80.3% of those in the chemotherapy arm manifested treatment-related adverse events (AEs), with incidences of 16.3% and 33.3% respectively for grade 3/4 treatment-related AEs, and 11.7% and 15.6% respectively for severe treatment-related AEs. In addition, there were three treatment-related deaths in the atezolizumab arm, compared to four with chemotherapy. The incidence of AEs requiring treatment discontinuation was similar in the two arms (13.0% with atezolizumab and 13.6% with chemotherapy), while AEs requiring treatment modification or interruption were less frequent with atezolizumab (32.0% versus 48.3%) [17].

The randomized phase III study EMPOWER-Lung 1 was designed to evaluate first-line treatment with cemiplimab (anti-PD-1) monotherapy versus standard chemotherapy (platinum-based doublet) in patients with advanced NSCLC with PD-L1 expression ≥ 50% (*n* = 563). The results of the study demonstrate a survival benefit of cemiplimab immunotherapy superior to that of chemotherapy and, in particular, the HR (95% CI) for OS in patients < 65 and ≥65 years was 0.66 (0.44–1.00) and 0.48 (0.30–0.76) respectively. In the intention-to-treat (ITT) population (*n* = 710), HR in patients younger and older than 65 was 0.72 (0.51–1.02) and 0.63 (0.43–0.91) respectively [18]. Table S2 lists the clinical trials with mono-immunotherapy for advanced NSCLC described.

Several non-randomised trials and real-world data studies have focused on elderly patients with NSCLC to assess the age impact on immunotherapy outcomes. In a retrospective analysis of 245 patients who received any line of immunotherapy agents, a trend towards numerically longer progression-free survival (PFS) was observed with increasing patient age, with peak PFS between 70 and 79 years. This trend did not extend to patients aged 80 years and older, who experienced the lowest median PFS, although this difference was not statistically significant. OS was similar among patients aged less than 60 years, 60–69 and 70–79 years (medians of 13.01, 14.56 and 12.92 months respectively). In contrast, patients aged 80 years and older had a shorter median OS (3.62 months) than patients in the other age groups, with an HR of 2.74 (*p* = 0.002) compared to younger patients [19]. In a further retrospective analysis of 928 geriatric patients undergoing immunotherapy alone, among NSCLC patients with documented response data (*n* = 276) ORR was 32.2%. ORR in NSCLC patients who were <85 and ≥85 years old was 34.5% and 25.7% respectively (*p* = 0.18). In the overall NSCLC cohort (*n* = 345), median PFS was 6.7 months (95% CI 5.2–8.6 months) and median OS 10.9 months (95% CI 8.6–13.1 months). In patients with NSCLC aged <85 and ≥85 years, median PFS was 8.0 (95% CI 5.6–9.5) and 5.0 (95% CI 4.0–8.4) months (*p* = 0.40) respectively. Median OS was 11.8 (95% CI 9.3–15.3) versus 7.5 (95% CI 5.0–11.5) months respectively (*p* = 0.047) [20]. A real-world study analyzed a large cohort of elderly patients with NSCLC who started therapy with ICIs and found no association between age and survival. Comorbidities, squamous histology, recent radiotherapy and time from diagnosis to treatment were found to be associated with a higher hazard of death [21]. Furthermore, a meta-analysis of seven randomized trials conducted by Khan et al. compared anti-PD-(L)1 monotherapy to chemotherapy in patients with advanced NSCLC. Immunotherapy agents led to a better OS (HR 0.72, 95% CI 0.63–0.82; *p* < 0.00001) and PFS (HR 0.84, 95% CI 0.72–0.97; *p* < 0.02) compared to chemotherapy. Age had no impact on PFS (patients under 65 years of age reached a better PFS but this difference was not significant), while an OS improved with immunotherapy was detected in patients over 65 (*p* = 0.006) but not in those older than 75 years (*p* = 0.56) [22]. Another meta-analysis on the efficacy of immunotherapy versus chemotherapy based on age (<65 versus ≥65) in NSCLC patients showed comparable survival between young and elderly patients (HR 0.75, 95% CI 0.64–0.88 versus 0.76, 95% CI 0.66–0.87) only in terms of OS [23]. Overall, mono-immunotherapy was not shown to be less effective in elderly advanced NSCLC patients than in younger ones.

### 3.2. Chemo-Immunotherapy and Other Combinations with Immune Checkpoint Inhibitors

Table S3 shows clinical trials with immunotherapy in combination regimens for advanced NSCLC. The combination of pembrolizumab and platinum-based chemotherapy was explored in two phase III clinical trials, KEYNOTE-189 and KEYNOTE-407, in non-treated patients with advanced non-squamous and squamous NSCLC respectively. PFS and OS were improved in patients aged less than 65 years and in those aged 65 years or older [24,25]. In the final analysis of the KEYNOTE-189 study, in the subgroup of participants ≥ 75 years, a HR of 1.54 (95% CI 0.76–3.14) in OS and a HR of 1.12 (95% CI 0.56–2.22) in PFS were derived for the combination with pembrolizumab compared to chemotherapy. In the same subgroup of the KEYNOTE-407 study, HR 0.81 (95% CI 0.43–1.55) in OS, HR 0.61 (95% CI 0.34–1.09) in PFS and ORR of 62% and 45% for the combination with pembrolizumab versus chemotherapy were reported. Atezolizumab was also combined with platinum-based chemotherapy in the phase III IMpower 150 clinical study, which evaluated the efficacy of the drug in association with carboplatin, paclitaxel and bevacizumab-based chemotherapy in patients with untreated advanced NSCLC, including cancers with epidermal growth factor receptor (EGFR) or anaplastic lymphoma kinase (ALK) alterations [26]. The study included 149 patients (37.2%) aged 65–74 years, 33 (8.2%) aged 75–84 years and 3 (0.8%) aged ≥85 years. The primary endpoint was met, and longer PFS was achieved in the ITT population without molecular alterations (8.3 versus 6.8 months; HR 0.62, 95% CI 0.52–0.74). HR was 0.52 and 0.78 in patients aged 65–74 years and ≥75 years respectively, with a median PFS of 9.7 months in the experimental arm and 6.9 months in the control arm [26]. In the updated analysis, median OS in the ITT wild-type population was 19.5 months with the four-drug regimen versus 14.7 months with the same regimen without atezolizumab (HR 0.80, 95% CI 0.67–0.95). In ITT patients, median OS was 19.8 months in the four-drug group, against 15.0 months in the no-immunotherapy three-drug group. The four-drug combination also gave an OS benefit in the 65–74 age subgroup (but not in patients over 75 years of age), though this analysis was limited by the low number of patients [27]. IMpower 130 is a phase III randomized study in chemotherapy-naive patients with metastatic non-squamous NSCLC, which assessed the efficacy of atezolizumab in combination with platinum-based chemotherapy versus chemotherapy alone. In the study evaluating atezolizumab combined with chemotherapy, subgroup analysis by age from the OS analysis showed a HR of 0.78 (95% CI 0.58–1.05) in patients ≥ 65 years [28]. In the first-line phase III IMpower 131 study of atezolizumab plus chemotherapy in patients with squamous cell lung cancer, no OS advantage was observed in elderly patients [29,30].

The Food and Drug Administration (FDA) recently approved a new indication for cemiplimab combined with chemotherapy as a first-line treatment for patients with advanced NSCLC, regardless of PD-L1 expression or tumor histology [31]. The new approval is based on a randomized phase III study, EMPOWER-Lung 3, conducted on 466 patients of whom 128 (41.0%) were ≥65 years old and received cemiplimab plus chemotherapy, while 60 (39.0%) were also ≥ 65 years old and received placebo plus chemotherapy. Treatment with cemiplimab plus platinum-based chemotherapy achieved a statistically significant improvement in OS (primary endpoint) in comparison to placebo plus chemotherapy (HR 0.71, 95% CI 0.53–0.93; *p* = 0.014), with 21.9 months (95% CI 15.5-not evaluable (NE)) survival versus 13.0 months (95% CI 11.9–16.1). With regard to OS according to subgroups, a HR of 0.57 (95% CI 0.40–0.81) and 0.88 (95% CI 0.56–1.37) was reported in patients under 65 and over 65 years respectively. Median PFS was 8.2 months (95% CI 6.4–9.3) in the experimental arm and 5.0 months (95% CI 4.3–6.2) in the control arm (HR 0.56, 95% CI 0.44–0.70; *p* < 0.0001). With regard to PFS according to subgroups, a HR of 0.53 (95% CI 0.39–0.71) and 0.56 (95% CI 0.39–0.81) was reported in patients under 65 and over 65 years respectively [32].

In the phase III CheckMate 227 trial, treatment-naive patients with advanced NSCLC without molecular drivers were randomized to receive a combination of nivolumab plus ipilimumab (anti-cytotoxic T lymphocyte-associated antigen 4 (CTLA-4)), standard chemotherapy, or nivolumab as monotherapy. The study analysis focused on the comparison between the combination arm and the standard chemotherapy arm. The study achieved both primary independent endpoints: PFS with nivolumab-ipilimumab compared to chemotherapy, in patients whose tumors had a high tumor mutational burden (TMB) (≥10 mut/Mb), independently of PD-L1 expression; and OS, showing a superior benefit for nivolumab-ipilimumab compared to chemotherapy in advanced NSCLC patients whose tumors expressed PD-L1 ≥ 1% [33]. Less than 10% of the study population was represented by patients older than 75 years; 81 patients with PD-L1 ≥1% were evaluated for OS: a HR of 0.93 (95% CI 0.59–1.49) was obtained from nivolumab combined with ipilimumab versus chemotherapy [33,34,35]. CheckMate 9LA is a phase III trial in which naive patients with advanced NSCLC and no molecular drivers were randomized to receive the combination nivolumab plus ipilimumab plus two cycles of standard platinum-based chemotherapy, or standard platinum-based chemotherapy alone, regardless of PD-L1 expression and histology. With 12.7 months minimum follow-up, combination immunotherapy improved OS compared with chemotherapy alone (median OS 15.6 versus 10.9 months, respectively; HR 0.66, 95% CI 0.55–0.80). Clinical benefit was observed in efficacy assessments in population subgroups such as patients aged 65–75 years (*n* = 295, 41%), where the HR was similar to that in patients aged less than 65 years (HR 0.61, 95% CI 0.47–0.80), but not in patients older than 75 years (HR 1.21, 95% CI 0.69–2.12) [36].

In the randomized phase III POSEIDON trial, patients with advanced NSCLC were randomized into three arms to receive tremelimumab (anti-CTLA-4) plus durvalumab (anti-PD-L1) plus chemotherapy; durvalumab plus chemotherapy; or chemotherapy alone as first-line treatment. On the basis of the findings of this study, the FDA has also recently approved durvalumab in combination with tremelimumab and platinum-based chemotherapy for the first-line treatment of advanced-stage NSCLC patients [37]. In the comparison durvalumab plus tremelimumab plus chemotherapy versus chemotherapy alone, PFS was significantly improved by the triple therapy (HR 0.72, 95% CI 0.60–0.86; *p* = 0.0003; median PFS 6.2 versus 4.8 months) [38]. Unlike the Mystic study, in which tremelimumab did not prolong survival, OS was significantly improved (HR 0.77, 95% CI 0.65–0.92; *p* = 0.0030; median OS 14.0 versus 11.7 months) when comparing triple therapy versus chemotherapy [38,39]. However, the primary endpoint of the study was the evaluation of the addition of durvalumab, and the evaluation of the addition of the immunotherapy doublet to chemotherapy was “relegated” to a secondary endpoint.

Additionally, countless retrospective studies have been carried out to evaluate treatment with immunotherapy along with chemotherapy in NSCLC patients. The combination of pembrolizumab with platinum and pemetrexed, and with carboplatin and (nab)paclitaxel, was studied in 122 and 81 patients respectively, in an analysis in which 43 patients (21.2%) were aged 75 years and older. PFS and OS were lower than in younger patients in the pemetrexed treatment group while there were no significant differences in PFS and OS between older and non-older patients in the paclitaxel treatment group [40]. A large real-world study on first-line chemo-immunotherapy versus mono-immunotherapy in NSCLC patients with advanced disease showed a median OS of 10.6 months (95% CI 9.3–11.8) for squamous carcinoma and 12.0 months (95% CI 11.3–12.8) for non-squamous carcinoma. Relative to squamous histotype, a longer median OS (95% CI) was achieved in patients aged 65–74 years compared to those aged <65 years or ≥75 years: 14.5 versus 8.9 versus 9.3 months respectively. In the non-squamous histotype, a shorter median OS (95% CI) of 10.1 months was found in patients with age range ≥ 75 years; values for patients aged 65–75 years and those below 65 years were 12.3 and 13.2 months respectively [41]. In a systematic review and meta-analysis by Zhang et al. of 8176 patients with advanced lung cancer, subgroup analysis did not show a significant difference between the benefit obtained in OS with first-line ICIs in younger (HR 0.89, 95% CI 0.71–1.12) and older (HR 0.87, 95% CI 0.71–1.07) patients, using a cutoff of 65 years. Conversely, with a cutoff of 75 years, the subgroup analysis did not reveal a benefit from ICIs in older patients (*p* = 0.520) [42]. 4994 patients were evaluated in another meta-analysis: ICIs significantly extended OS (HR 0.73, 95% CI 0.61–0.89) compared to chemotherapy alone in NSCLC patients younger than 65 years. They also extended OS (HR 0.74, 95% CI 0.59–0.93) in patients with NSCLC who were older than 65 years of age. However, no statistical significance of OS was found (HR 0.87, 95% CI 0.57–1.30) among patients with NSCLC who were older than 75 [43]. Finally, Yan et al. evaluated the efficacy of immunotherapy in combination regimens for NSCLC in a systematic review and meta-analysis including 5487 patients. The result was a significant improvement in OS and PFS with ICIs-based combination therapy in younger as well as older patients compared to therapy without ICIs, given an age cutoff of 65 years [44]. Although combination chemotherapy and immunotherapy trials include small percentages of elderly patients, published data are somewhat mixed, and further exploration of the mechanism by which age affects combination therapy is needed, overall these patients do not appear to have little benefit from such an approach. It should, consequently, be proposed as a standard therapeutic option whenever possible, especially in so-called young elderly patients (<75 years).

## 4. Immunotherapy Toxicity in Elderly Patients

Safety is of particular importance for elderly patients, given their potential drug tolerability issues due to reduced renal function, cardiac or other comorbidities, deteriorating organ function and impaired cognitive ability. There are few clinical trials reporting AEs in relation to age. Atezolizumab has recently been shown to stabilize and/or improve some measures of Health-Related Quality of Life (HRQoL), and no new or unexpected safety problems have been reported in the study population of the IPSOS trial [17]. In the pooled analysis of the KEYNOTE-010, -024 and -042 studies, pembrolizumab was associated with a lower number of treatment-related AEs than chemotherapy in elderly patients aged ≥75 years (overall, 68.5% vs. 94.3%; grade ≥ 3, 24.2% vs. 61.0%), and the results observed in these patients were comparable to those of the overall populations in the individual studies [14]. Single-agent immunotherapy in older cancer patients did not correlate with an increased occurrence of high-grade immune toxicity in the ELDERS study, the first prospective study planned to investigate the safety of immunotherapy in older cancer patients [45]. This was an observational study with two cohorts, ≥70 and <70 years; eligible patients were those with advanced NSCLC or melanoma starting single-agent immunotherapy. No significant difference was found between the incidence of grade 3–5 immune-related adverse events (irAEs) in the ≥70 and <70 years cohorts (18.6% versus 12.9%; odds ratio 1.55, 95% CI 0.61–3.89; *p* = 0.353) [45]. The toxicity of single-agent immunotherapy in cancer patients aged 80 years and older was retrospectively analyzed in 928 patients, including 345 with NSCLC. 113 patients (12.2%) experienced G3-G4 irAEs. Patients in each age subgroup developed irAEs of any grade at similar rates. 137 patients (16.1%) interrupted treatment, particularly those ≥90 years old (30.9% versus 15.1% for younger patients) [45]. In another retrospective analysis of 245 NSCLC patients who received PD-1/PD-L1 inhibitors, 102 (41.6%) experienced an irAE, with no age differences (*p* = 0.652) [19]. Furthermore, a retrospective study explored the association of age with the emergence of irAEs in 527 patients with NSCLC, treated with pembrolizumab or nivolumab. 214 (40.6%) patients were aged ≤64 years, 214 (40.6%) were aged 65–74 years and 99 (18.8%) were aged 75 years or older. No difference was detected between the age groups as regards irAEs of any grade (*p* = 0.98), but interruption because of irAEs at 6 weeks was more common in patients aged 75 years and older (*p* = 0.055) [46]. Finally, no toxicity problems emerged even among older patients in a retrospective analysis of 290 NSCLC patients of whom 110 (38%) were more than 70 years old (*p* = 0.6493) [47]. According to what has been described so far, while toxicity is comparable in terms of incidence of high-grade AEs and irAEs in NSCLC patients in the various age subgroups who receive ICIs as single agents, the discontinuation rate due to irAEs appears more common as age increases.

As concerns the age-based safety of previously untreated NSCLC patients, clinical trials with ICIs in combination regimens lack this assessment [24,25,26,28,29]. In the retrospective analysis by Morimoto et al. of the clinical impact of age in NSCLC patients receiving combination regimens of pembrolizumab plus chemotherapy, patients ≥ 75 years of age were more affected by treatment-related grade ≥ 3 AEs than those <75 years of age, although the difference was not statistically significant. Specifically, the incidences of non-haematologic and hematologic AEs with the pemetrexed and platinum chemotherapy combination were 36.0% versus 26.8% (*p* = 0.46) and 32.0% versus 26.8% (*p* = 0.62) respectively, while the corresponding values for the combination of taxane and platinum chemotherapy were 27.8% versus 28.6% (*p* = 1.0) and 55.6% versus 30.2% (*p* = 0.09). Grade 3–5 immune-related pneumonias were reported with a higher incidence in older patients treated with pembrolizumab and pemetrexed (16.0% versus 2.1%, *p* = 0.02) [40]. Fujimoto et al. retrospectively evaluated chemo-naive patients with advanced NSCLC who were given a combination of platinum, pemetrexed and pembrolizumab. 299 patients were included, of whom 43 (14%) were elderly. The severe AEs rate was higher in the elderly than the younger patients (26% versus 19%, *p* = 0.312). The AEs-related treatment discontinuation rate was significantly higher in the elderly (40% versus 21%, *p* = 0.012). Another safety analysis in patients aged <65, 65–74 and ≥75 years resulted in higher rates of severe AEs with higher age (16%, 21% and 26% respectively); results for treatment discontinuation rates showed the same pattern (14%, 27% and 40% respectively) [48].

## 5. Discussion

The treatment of NSCLC has markedly improved over recent decades with the introduction of new active drugs into clinical practice and the combination of various therapeutic modalities. In particular, immunotherapy has totally changed the therapeutic algorithm. NSCLC is primarily considered a disease of the elderly, but there is a paucity of evidence to guide treatment decisions in this heterogeneous population. These individuals, and especially the frail elderly, are notoriously underrepresented in clinical trials. Screening tools may facilitate the identification of patients who, being at higher risk of vulnerability or frailty, deserve a comprehensive geriatric assessment (CGA). This, in turn, may guide therapeutic orientation by identifying unacknowledged aspects, thus revealing a high risk of treatment toxicity, patient resilience and potential interference with treatment efficacy [49,50]. The screening function of the G8 scale and the CGA was investigated in the prospective ELDERS study, which informed on the role of these tools in predicting the occurrence of irAEs in patients undergoing immunotherapy [45]. Patients with a positive G8 screening had a baseline CGA and, overall, had worse ECOG PS and a higher polypharmacy and comorbidity burden than patients > 70 years of age with a negative score on G8 screening. A positive G8 screening was found to correlate with a higher rate of hospitalization and a higher risk of death. No definitive conclusions could be drawn regarding the impact of CGA on treatment results [45]. While on the one hand screening tools for geriatric assessment can help to select patients and personalize therapies, while preventing undertreatment of fit elderly patients, on the other there are few studies that have used these scales, whose role in trials with ICIs remains still limited. Examining the particularities of elderly patients with cancer is of great interest in order to define potential response factors to ICIs in this population. Immunosenescence is the immune dysfunction associated with aging, and involves innate and specific immunity. It is characterized by an increased ratio of memory T cells to naive T cells; an increased proportion of circulating senescent T cells having low proliferative activity and associated with defined phenotypic markers; an oligoclonal T cell receptor repertoire; a reduced capacity of immune cells to recognise and bind antigens; and an overall increase in the pro-inflammatory state resulting in an increase in immunosuppressive regulatory T cells and a state of chronic low-grade inflammation that occurs with advancing age (commonly known as “inflammaging”) [51,52,53,54]. Ferrara et al. evaluated a “senescent immune phenotype” by measuring the percentage of CD28−CD57+KLRG-1+ cells among circulating CD8+ T lymphocytes from patients with NSCLC treated with ICIs. They found that a percentage of >39.5% senescent immune phenotype (SIP)+ cells was associated with reduced efficacy of the single-agent PD-1/PD-L1 inhibitors, but baseline SIP status was not significantly associated with chronological age [55]. The composition of the microbiome is also linked to the functioning of the immune system, and the reduced microbiota diversity associated with aging may hinder the efficacy of ICIs [56,57]. Other aspects characteristic of advanced age to be considered are the lack of DNA repair capacity and the metabolic changes that contribute to local inflammation, cancer escape from the immune system and metastasis [58,59,60,61]. Further research and a deeper understanding of the described processes may help generate potential biomarkers of response to ICIs in this population, such as the Lung Immune Prognostic Index (LIPI). A poor LIPI score has been associated with worse outcome in older patients treated with anti-PD-(L)1, providing an example of how such a tool could be useful for stratifying the benefits of immunotherapy [62]. Clinical trials focusing on elderly patients with advanced NSCLC are currently underway to compare immunotherapy plus chemotherapy with immunotherapy alone, to evaluate an alternative chemotherapy backbone to be combined with ICIs, and to test different treatment sequences: NCT03977194 (ELDERLY), NCT03975114 (MILES-5), NCT04533451, NCT03345810 (DURATION).

At present, age per se is not a limitation for treatment selection, but should be considered as a surrogate for other potentially age-related factors (ECOG PS, comorbidities, etc.). Geriatric assessment is more effective than PS in identifying frail patients at increased risk of hospitalization and death. Frail patients should be under the care of a geriatrician, who should be part of the multidisciplinary team, so that corrective interventions can be taken. While potentially not an issue for the single anti-PD-1/PD-L1 agent, the toxicity of chemotherapy components raises no shortage of concerns for combination chemotherapy plus immunotherapy in octogenarians with advanced NSCLC. Basically, the available data are exploratory analyses from clinical trials with no differences in efficacy seen at the cutoff age of 65 years, while only a few elderly patients (≥75 years) were included in the trials. The use of dual immunotherapy with anti-CTLA4 plus anti-PD-1 in combination with chemotherapy, employing the regimen with only 2 cycles of chemotherapy without maintenance pemetrexed, could be favorable in the elderly population. Undoubtedly, the treatment of advanced lung cancer in the elderly remains challenging for a number of reasons. With increasing life expectancy and accessible treatment options, geriatric oncology will be an increasing area of interest which will have to deal with a population with a multitude of molecular and immune changes and a preponderance of chronic-degenerative diseases. Immunotherapy produces lasting benefits even in the elderly, in whom it has been shown to be feasible and safe, but data in patients older than 75 are particularly lacking. It is highly desirable to define clinical and biological predictive factors of response, based for example on immunocompetence, which allow a better understanding of the immunotherapy-resistance phenomenon and ensure the most appropriate treatment for each individual patient. Real-world supplementation of elderly patients not included in clinical trials, real-world assessment of specific subsets of elderly patients (frail, with comorbidities) and better data quality from real-world databases to assess all the subsets are, among others, valid recommendations for clinical research. Figure S1 proposes a therapeutic algorithm for the first-line treatment of advanced NSCLC in the elderly, based on data collected to date from both randomized and retrospective clinical trials, with the proviso that individual patient characteristics (comorbidities, concomitant medications, etc.) and periodic toxicity assessments to improve treatment adherence and tolerance should always be considered from the outset. Dose and schedule adjustments and close monitoring during treatment may be required, especially for the vulnerable elderly, who should be considered for adapted treatment such as single agent chemotherapy followed by single agent ICI for patients with PD-L1 TPS <50%. Overall, treatment decisions should take into account the patient’s overall health status (including the family and social contexts) and treatment goals while respecting the patient’s expressed will.

## 6. Conclusions

In light of what has been said so far, in the unselected elderly patient, frequently affected by comorbidity and frailty, a therapeutic choice that takes into account the balance between efficacy, disease control and risk of toxicity remains crucial. Recent findings from a large cohort of patients with advanced NSCLC demonstrate substantially different survival gains depending on age in a study aimed at assessing improvements in survival in the era of ICIs using clinical practice data [63]. There are no conclusive data about the higher risk of severe irAEs in the elderly treated with immunotherapy compared to younger patients, although there are conflicting results related to the heterogeneity of the study populations as well as a tendency towards autoimmunity in senior patients [64,65,66,67]. Some interesting studies have verified the role of concomitant drugs during immunotherapy, although wider prospective studies are needed to identify which drugs should be avoided. One study has presented the outcome analysis depending on concomitant drugs at baseline in a large cohort of patients with metastatic NSCLC (PD-L1 expression ≥ 50%) undergoing first-line monotherapy with pembrolizumab; patients undergoing chemotherapy represented the control cohort [68]. On multivariable analysis, antibiotics were a strong indicator of worse OS and PFS only in the pembrolizumab cohort; corticosteroids were associated with shorter PFS and OS in both cohorts; proton pump inhibitors were associated with worse OS with pembrolizumab and chemotherapy [68]. A prognostic score based on the aforementioned drugs was validated, which was able to stratify NSCLC patients who were candidates for pembrolizumab monotherapy [69,70]. A Japanese retrospective study evaluated the efficacy of ICIs in patients aged ≥65 years with advanced NSCLC; more than half of them were taking polytherapy, defined as ≥5 drugs. Median survival in patients with and without polytherapy was 9.5 and 28.1 months respectively (*p* < 0.001). Multivariate analysis revealed a strong association between polypharmacy and OS. Polypharmacy was also associated with a higher rate of hospitalisations during treatment with ICIs but was not associated with irAEs [71]. Prospective clinical trials specifically designed for elderly patients enrolled not only on the basis of age but also and above all after a global geriatric analysis that includes the evaluation of functionality and quality of life, particularly in the frail or chronically ill, remain a decisive topic to address. It is assumed in any case that "fit" elderly patients should be treated in the same way as their younger counterparts.

## Supplementary Materials

The following supporting information can be downloaded at: https://www.mdpi.com/article/10.3390/cancers15082319/s1, Table S1: A. The KEYNOTE-010, KEYNOTE-024 and KEYNOTE-042 pooled analysis: overall survival following treatment with pembrolizumab or chemotherapy by patient age (≥75 or <75 years) in the population with PD-L1 TPS ≥ 1% or ≥50% (a) and in an analysis of patients from each individual trial (b). B. FDA pooled analysis of randomized controlled trials with anti-PD-(L)1 combined with chemotherapy versus immunotherapy alone for first-line treatment of advanced NSCLC with PD-L1 score 1–49%; Table S2: Overall survival by age of patients in randomized phase III clinical trials with mono-immunotherapy for advanced stage NSCLC; Table S3: Overall survival by age of patients in randomized phase III clinical trials with immunotherapy in combination regimens for advanced stage NSCLC; Figure S1: Therapeutic algorithm for the first-line treatment of elderly advanced NSCLC.
