# Supplementary material for: The Role of Immunotherapy in the First-Line Treatment of Elderly Advanced Non-Small Cell Lung Cancer
Source: Cancers (Basel). 2023 Apr 15;15(8):2319. doi: 10.3390/cancers15082319 (PMC10136482; doi:10.3390/cancers15082319)
Supplement: Supplementary file 1 [file cancers-15-02319-s001.zip › cancers-2222363-supplementary.pdf]

cancers

The role of immunotherapy in the first-line treatment of elderly advanced non-small cell lung cancer

Alessia Spagnuolo and Cesare Gridelli

Table S1. A. The KEYNOTE-010, KEYNOTE-024 and KEYNOTE-042 pooled analysis: overall survival following treatment with pembrolizumab or chemotherapy by patient age (≥75 or <75 years) in the population with PD-L1 TPS ≥1% or ≥50% (a) and in an analysis of patients from each individual trial (b). B. FDA pooled analysis of randomized controlled trials with anti-PD-(L)1 combined with chemotherapy versus immunotherapy alone for first-line treatment of advanced NSCLC with PD-L1 score 1-49%.

**1A**		
**a**	**Overall survival by age HR (95% CI)**	
	**Patients ≥ 75 years**	**Patients < 75 years**
**PD-L1 TPS ≥1%**	0.76 (0.56-1.02)	0.76 (0.69-0.84)
**PD-L1 TPS ≥50%**	0.40 (0.25-0.64)	0.67 (0.57-0.78)
**b**	**Overall survival by age HR (95% CI)**	
	**Patients ≥ 75 years**	**Patients < 75 years**
**KEYNOTE-010**	0.72 (0.43-1.21)	0.64 (0.55-0.75)
**KEYNOTE-024**	0.49 (0.17-1.39)	0.64 (0.42-0.97)
**KEYNOTE-042**	0.89 (0.59-1.35)	0.79 (0.68-0.92)

**1B**		
**Overall survival by age HR (95% CI)**		
**Patients <65 years**	**Patients 65-74 years**	**Patients ≥75 years**
0.63 (0.43-0.92)	0.61 (0.38-0.97)	0.95 (0.42-2.14)

Abbreviations. CI: confidence interval; HR: hazard ratio; PD-L1 TPS: programmed death-ligand 1 tumor proportion score

Table S2. Overall survival by age of patients in randomized phase III clinical trials with mono-immunotherapy for advanced stage NSCLC.

**Study**	**Treatment line**	**PD-L1 expression**	**Intervention/arms**	**Overall survival by age HR (95% CI)**
**KEYNOTE-024**	First-line	PD-L1≥50%	Pembrolizumab vs platinum-based chemotherapy	<65: 0.60 (0.38-0.96)<br>≥65: 0.64 (0.42-0.98)
**KEYNOTE-042**	First-line	PD-L1≥1%	Pembrolizumab vs platinum-based chemotherapy	<65: 0.81 (0.67-0.98)<br>≥65: 0.82 (0.66-1.01)
**IMpower 110**	First-line	PD-L1≥1%	Atezolizumab vs platinum-based chemotherapy	<65: 0.59 (0.34-1.04)<br>65-74: 0.63 (0.34-1.19)<br>≥75: 0.79 (0.18-3.56)
**IPSOS**	First-line	Stratification factor	Atezolizumab vs vinorelbine or gemcitabine	0.78 (0.63-0.97)<br><70: 0.75 (0.49-1.14)<br>70-79: 0.68 (0.49-0.94)<br>≥80: 0.97 (0.66-1.44)
**EMPOWER-Lung 1**	First-line	PD-L1≥50%	Cemiplimab vs platinum-based chemotherapy	<65: 0.66 (0.44-1.00)<br>≥65: 0.48 (0.30-0.76)

Abbreviations. CI: confidence interval; HR: hazard ratio; PD-L1: programmed death-ligand 1.

Table S3. Overall survival by age of patients in randomized phase III clinical trials with immunotherapy in combination regimens for advanced stage NSCLC.

Study	Treatment line	Intervention/arms	Overall survival by age HR (95% CI)
**KEYNOTE-189**	First-line	Platinum-based chemotherapy + pembrolizumab or placebo	<65: 0.43 (0.31-0.61)<br>≥65: 0.64 (0.43-0.95)<br>≥75: 1.54 (0.76-3.14)
**KEYNOTE-407**	First-line	Platinum-based chemotherapy + pembrolizumab or placebo	<65: 0.52 (0.34-0.80)<br>≥65: 0.74 (0.51-1.62)<br>≥75: 0.81 (0.43-1.55)
**IMpower 150**	First-line	Platinum-based chemotherapy and bevacizumab +/- atezolizumab	<65: 0.83 (0.65-1.04)<br>65-74: 0.72 (0.54-0.97)<br>≥75: 0.97 (0.58-1.62)
**IMpower 130**	First-line	Platinum-based chemotherapy +/- atezolizumab	<65: 0.79 (0.58-1.08)<br>≥65: 0.78 (0.58-1.05)<br>75-84: 0.54 (0.27-1.05)
**IMpower 131**	First-line	Platinum-based chemotherapy +/- atezolizumab	<65: 0.89 (0.68-1.15)<br>65-74: 0.84 (0.63-1.13)<br>≥75: 0.74 (0.45-1.23)
**EMPOWER-Lung 3**	First-line	Platinum-based chemotherapy + cemiplimab or placebo	<65: 0.57 (0.40-0.81)<br>≥65: 0.88 (0.56-1.37)
**CheckMate 227**	First-line	Nivolumab + ipilimumab vs platinum-based chemotherapy	PD-L1 ≥1%:<br><65: 0.70 (0.56-0.89)<br>65-74: 0.84 (0.65-1.08)<br>≥75: 0.93 (0.58-1.49)<br>PD-L1 <1%:<br><65: 0.69 (0.51-0.93)<br>65-74: 0.57 (0.39-0.84)<br>≥75: 0.74 (0.32-1.71)
**CheckMate 9LA**	First-line	Platinum-based chemotherapy + nivolumab + ipilimumab vs platinum-based chemotherapy	<65: 0.61 (0.47-0.80)<br>65-74: 0.62 (0.46-0.85)<br>≥75: 1.21 (0.69-2.12)
**POSEIDON**	First-line	Tremelimumab + durvalumab + platinum-based chemotherapy and durvalumab + platinum-based chemotherapy vs chemotherapy alone	<65: 0.79 (0.62-1.00)<br>≥65: 0.74 (0.58-0.94) and<br><65: 0.86 (0.68-1.10)<br>≥65: 0.81 (0.64-1.03)

Abbreviations. CI: confidence interval; HR: hazard ratio; PD-L1: programmed death-ligand 1.

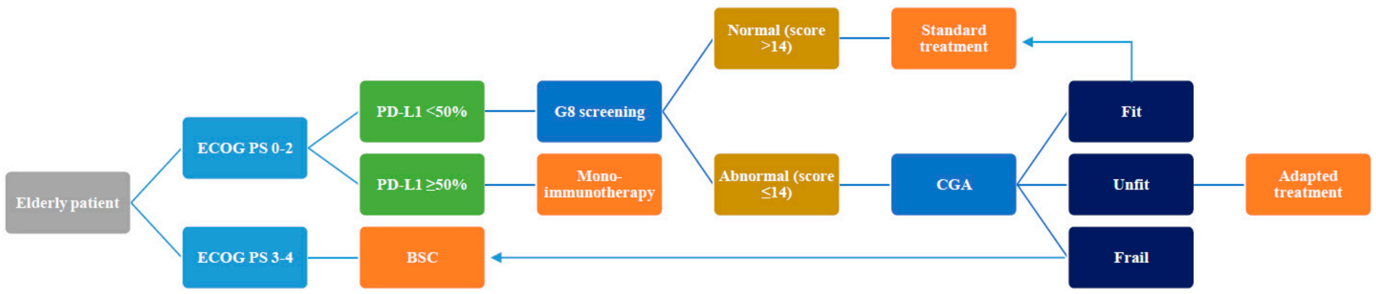

Figure S1. Therapeutic algorithm for the first-line treatment of elderly advanced NSCLC. Abbreviations: BSC: Best Supportive Care; CGA: Comprehensive Geriatric Assessment; ECOG PS: Eastern Cooperative Oncology Group Performance Status; PD-L1: Programmed death-ligand 1.
